# Faecal colonization of *E. coli* and *Klebsiella* spp. producing extended-spectrum beta-lactamases and plasmid-mediated AmpC in Mozambican university students

**DOI:** 10.1186/s12879-018-3154-1

**Published:** 2018-05-30

**Authors:** L. M. Chirindze, T. F. Zimba, J. O. Sekyere, U. Govinden, H. Y. Chenia, A. Sundsfjord, S. Y. Essack, G. S. Simonsen

**Affiliations:** 1 Microbiology Laboratory, Maputo Central Hospital, Maputo, Mozambique; 2 High Institute of Health Sciences (ISCISA), Maputo, Mozambique; 3 Antimicrobial Research Unit, School of Health Sciences, University of KwaZulu-Natal, Durban, South Africa; 4 Discipline of Microbiology, School of Life Sciences, University of KwaZulu-Natal, Durban, South Africa; 5 Research Group for Host-Microbe Interaction, Institute of Medical Biology, Faculty of Health Sciences, UiT – Arctic University of Norway, Tromsø, Norway; 6 Department of Microbiology and Infection Control, Norwegian National Advisory Unit on Detection of Antimicrobial Resistance, University Hospital of North Norway, 9038 Tromsø, Norway

**Keywords:** *E. coli*, *Klebsiella*, ESBL, pAmpC, Colonization, Students, Mozambique

## Abstract

**Background:**

In recent years, the world has seen a surge in Enterobacteriaceae resistant to broad-spectrum beta-lactam antibiotics due to the production of extended-spectrum beta-lactamases (ESBLs) or plasmid-mediated AmpC (pAmpC) enzymes. Data on the epidemiology of cephalosporin-resistant Enterobacteriaceae in Sub-Saharan Africa are still limited**.**

**Methods:**

Two hundred seventy-five non-repetitive stool samples were collected from Mozambican university students of both sexes. Samples were cultured on MacConkey agar with and without ceftriaxone (1 mg/L) for selection of third-generation cephalosporin-resistant isolates, which were subjected to antimicrobial susceptibility testing by disc diffusion, characterization of resistance genes by PCR and ERIC-PCR analysis for strain clonality.

**Results:**

Among the 275 students, 55 (20%) carried a total of 56 *E. coli* (*n* = 35) and *Klebsiella* spp. (*n* = 21) isolates resistant to ceftriaxone and phenotypically positive for ESBL- and/or pAmpC-production. Forty-three percent of the isolates (24/56) contained only ESBL genes, 11% (6/56) only pAmpC genes, and 36% (20/56) both ESBL and pAmpC genes. The remaining six isolates were negative for the CTX-M/pAmpC genes included in the test panel. *E. coli* and *Klebsiella* spp. combined demonstrated 70% resistance to tetracycline and co-trimoxazole, 63% to ceftazidime and 34% to ciprofloxacin. In total, 89% of ESBL/pAmpC-positive isolates were defined as multi-resistant by being resistant to three or more antibiotic classes. ERIC-PCR fingerprinting demonstrated low similarity among isolates. None of the participants reported recent hospitalization and just 12.5% had taken antibiotics 3 months prior to the study.

**Conclusion:**

This study demonstrated 20% colonization with multi-resistant *E. coli* and *Klebsiella* spp. among Mozambican students with a diversity of ESBL and pAmpC genes. Colonization was not related to prior hospitalization or antimicrobial consumption.

**Electronic supplementary material:**

The online version of this article (10.1186/s12879-018-3154-1) contains supplementary material, which is available to authorized users.

## Background

In recent years, the world has seen a surge in Enterobacteriaceae resistant to broad-spectrum beta-lactam antibiotics attributed to the production of extended-spectrum beta-lactamases (ESBLs) and/or plasmid-mediated AmpC (pAmpC) enzymes [1, 2]. ESBLs are, by the classical definition, inhibited by clavulanic acid [3] whereas pAmpC enzymes are not [4]. The plasmid location of ESBL and pAmpC genes facilitates their spread via horizontal gene transfer [5, 6].

*Escherichia coli* and *Klebsiella pneumoniae* are not only constituents of the commensal gut flora but also common opportunistic pathogens often implicated in urinary tract and bloodstream infections [7, 8]. They frequently harbor ESBL- and pAmpC-encoding genes. Broad-spectrum beta-lactamase production is associated with increased morbidity and mortality in both high- and low/middle-income countries [9, 10].

Data on the epidemiology of ESBL- and pAmpC-producing Enterobacteriaceae in Africa are still limited. The majority of publications report on the prevalence of ESBL-producing Enterobacteriaceae in clinical samples, and there are only few studies on colonization [11, 12]. Furthermore, there is a predominance of reports from Northern and Western Africa [13, 14] and only individual studies from Eastern and Southern Africa [15–17] (excluding South-Africa). The aim of this study was to determine the prevalence of fecal colonization with ESBL- and pAmpC-producing *E. coli* and *Klebsiella* spp. among healthy university students in Maputo, Mozambique, and to analyze the resistance phenotype, ESBL- and pAmpC resistance gene content and clonal relatedness of isolates.

## Methods

### Study sample

A random sample of 275 university students of both sexes living in three residences at the Eduardo Mondlane University in Maputo, Mozambique, provided non-repetitive stool specimens within a six-week period, from January to February 2016 upon informed, voluntary consent. Students with diarrheal disease were excluded from the study. Participants provided information about antibiotic use within 3 months and hospitalization within 6 months prior to the study. Stool samples were kept on ice and transported to the laboratory for immediate culture twice daily.

### Identification and susceptibility testing

All samples were cultured on MacConkey agar with and without ceftriaxone (1 mg/L) for selection of third-generation cephalosporin-resistant isolates. Lactose-positive isolates growing on the 1 mg/L ceftriaxone agar were identified to the species level using API20E (bioMérieux, South Africa). A total of 56 *E. coli* (*n* = 35) and *Klebsiella* spp. (*n* = 21) isolates were identified by this procedure. Two isolates were recovered from the same sample. These putative ESBL- and/or pAmpC-producers constituted the study sample and were subjected to antimicrobial susceptibility testing by disc diffusion to the following antibiotics: cefoxitin, ceftazidime, ceftriaxone, imipenem, amikacin, gentamicin, ciprofloxacin and co-trimoxazole. The results were interpreted according to the CLSI breakpoints to determine their susceptibility profile [18]. Multi-resistance was defined as resistance to three or more antibiotic classes. *E. coli* ATCC 25922 (wild-type) and *K. pneumoniae* ATCC 700603 (ESBL positive) were used as negative and positive quality control strains for antimicrobial susceptibility testing and ESBL screening, respectively.

### Phenotypic and genotypic characterization of beta-lactamases

ESBL production was confirmed by double disc synergy using ROSCO discs (Rosco Diagnostic, Taastrup, Denmark) containing ceftazidime, ceftriaxone and amoxicillin/clavulanic acid. pAmpC production was determined using ROSCO discs containing cefotaxime, cefotaxime+boronic acid, ceftazidime and ceftazidime+boronic acid [18]. Isolates with reduced susceptibility to carbapenems were subjected to the Carba NP test as described previously [19]. *E. coli* ATCC 25922 and *K. pneumoniae* ATCC 700603 were used as negative and positive quality control strains, respectively.

For DNA extraction, 18–24 h colonies grown on Muller Hinton agar were inoculated into Luria-Bertani (LB) broth and incubated at 37 °C with shaking [20]. After 20 h of incubation, DNA was extracted using the ZR Fungal/Bacterial DNA MiniPrep kit (Zymo Research, Lithuania). PCR amplifications for detection of *bla*_TEM_, *bla*_SHV_, *bla*_CTX_, *bla*_CMY_, *bla*_DHA_, *bla*_FOX_ and *bla*_MOX_ (Table 1) were performed in a ThermalCycler T100™ (Bio-Rad, USA) with a final volume of 50 μL (25 μL of Master mix, 15 μL of water, 4 μL of each primer with a final concentration of 0.8 μM (Inqaba Biotechnology Industries, South Africa) and 2 μL of template DNA), with an initial denaturation temperature of 98 °C for 10 s, extension at 72 °C for 15 s and a final extension at 72 °C for 1 min. The annealing temperatures were as follows: *bla*_TEM_ 60 °C, *bla*_SHV_ 56 °C, *bla*_CTX_ 57 °C, *bla*_CMY_ 57 °C, and *bla*_DHA_, *bla*_FOX_ and *bla*_MOX_ 50 °C. PCR products were loaded on a 1.5% (*w*/*v*) agarose gel and visualized by UV transillumination (Bio-Rad ChemiDoc™MP System) after staining in 0.1 mg/mL Gel Red for 15 min. A random sample of 19 CTX-M PCR amplicons were sent to Inqaba Biotec (South Africa) for DNA sequencing. TEM and SHV amplicons were not sequenced.

**Table 1:** Primers used for amplification of ESBL genes

Target enzyme	Primers	Sequence (5′ to 3′)	Annealing temperature (ref)
TEM-1	TEMMF	AAA ATT CTT GAA GAC G	60 °C [29]
	TEMMR	TTA CCA ATG CTT AAT CA	
SHV	SHVMF	TTA ACT CCC TGT TAG CCA	56 °C [29]
	SHVMR	GAT TTG CTG ATT TCG CCC	
CTX-1	CTXMF	GGT TAA AAA ATC ACT GCG TC	57 °C [27]
	CTXMR	TTG GTG ACG ATT TTA GCC GC	
CMY	CMYMF	GAT TCC TTG GAC TCT TCA G	57 °C [28]
	CMYMR	TAA AAC CAG GTT CCC AGA TAG C	
FOX	FOXMF	CAC CAC GAG AAT AAC CAT	57 °C [28]
	FOXMR	ATG TGG ACG CCT TGA ACT	
DHA	DHAMF	AAC TTT CAC AGG TGT GCT GGG T	57 °C [28]
	DHAMR	CCG TAC GCA TAC TGG CTT TGC	
MOX	MOXMF	GCT GCT CAA GGA GCA CAG GAT	50 °C [28]
	MOXMR	CAC ATT GAC ATA GGT GTG GTG C	

### Genomic DNA isolation and ERIC-PCR analysis

Genomic DNA was isolated and purified using the GeneJet Genomic DNA Purification Kit (Thermo Scientific, USA). Antibiotic-susceptible *E. coli* ATCC 25922 and beta-lactam resistant SHV-18 *K. pneumoniae* ATCC 700603 were used as quality controls. The total PCR reaction volume was 10 μL, which contained 100 ng of template DNA, 50 pmol of each primer, 2.8 μL nuclease-free water and 5 μL of DreamTaq PCR Mastermix (2X) (Thermo Scientific). The primers ERIC 1 and ERIC 2 [21] were used. PCR conditions were as follows: 94 °C for 3 min, 30 cycles of 30 s of denaturation at 94 °C, 1 min of annealing at 50 °C, 8 min of extension at 65 °C, and a final elongation of 16 min at 65 °C, in an Applied Biosystems 2720 thermal cycler. The ERIC-PCR products were loaded onto 1% (*w*/*v*) agarose gels and subjected to electrophoresis at 80 V using 1 × TAE buffer. Amplification products were visualized by UV transillumination (Syngene, UK) after staining in 0.1 mg/mL ethidium bromide for 15 min. Genotypic variation was analyzed using the Gel Compare II version 6.0 software package (Applied Maths, Belgium) by Jacquard index and Unweighted Pair Group Method with Arithmetic Mean (UPGMA) cluster analysis to produce a dendrogram.

### Statistical analyses

Groups were compared by the Fischer Exact Test using the Epi Info StatCalc software version 7.2.2.6 (CDC, Atlanta, USA) with statistical significance defined as *p* < 0.05.

## Results

### Setting and samples

Among the 275 samples collected in the study, 159 (58%) were retrieved from male students and 116 (42%) from females in an age range from 19 to 32 years old. The students lived in separate blocks and/or floors for male and female students. Each floor had one kitchen where students could prepare their own food. There were students from different courses including, but not limited to Engineering, Medicine, Political Science, Biology and Sociology. All participants declared a history of non-hospitalization 6 months prior to the study and 87.5% had not consumed any antibiotics for at least 3 months.

### Frequency of *E. coli* and *Klebsiella* spp. ESBL colonization

In 55 of 275 non-repetitive stool samples 35 *E. coli* and 21 *Klebsiella* spp. grew on MacConkey agar containing 1 mg/L ceftriaxone, with one sample providing two isolates. The overall prevalence of ceftriaxone-resistant *E. coli* and *Klebsiella* spp. colonization was thus 20% (55/275). There was no statistically significant difference in the rate of colonization between females (*n* = 28, 24%) and males (*n* = 28, 17%). All samples displayed growth on the ceftriaxone-free growth control agar.

### Phenotypic analyses of antimicrobial susceptibility

All the 56 *E. coli* (*n* = 35) and *Klebsiella* spp. (*n* = 21) isolates were phenotypically confirmed as ESBL and/or pAmpC producers and showed high rates of resistance to tetracycline and co-trimoxazole (70%), ceftazidime (63%), cefoxitin (41%), and ciprofloxacin (34%). Two isolates displayed carbapenem zone diameters below the screening breakpoint for carbapenemase production, but were categorized as susceptible by the clinical breakpoint. They were subsequently negative in the Carba NP test and there were thus no carbapenemase producers (Table 2). Eighty-eight percent of the isolates were defined as multi-resistant. Antimicrobial susceptibility results are presented in the Additional file 1: Table S1.

**Table 2:** Antimicrobial resistance (%) of ESBL-producing *E. coli* and *Klebsiella* spp

Antibiotic	*E. coli* (*n* = 35)	*Klebsiella* spp. (*n* = 21)
Ceftriaxone	100	100
Ceftazidime	71	43
Gentamicin	14	43
Cefoxitin	46	33
Tetracycline	66	76
Co-trimoxazole	63	76
Ciprofloxacin	37	29
Imipenem	0	0

### ESBL gene identification by PCR and sequencing

The PCR results for CTX-M and pAmpC genes are summarized in Table 3. Among 56 isolates, 41% were positive for at least two genes, 32% were positive for at least three, and 23% were positive for at least four genes. Forty-three percent (24/56) of the isolates contained only CTX-M, 11% (6/56) only pAmpC, and 36% (20/56) both CTX-M and pAmpC sequences. Six isolates (11%) were negative for CTX-M and pAmpC genes but contained TEM and/or SHV sequences (data not shown). However, as these amplicons were not sequenced it cannot be concluded that they encode broad-spectrum beta-lactamases and the basis for ceftriaxone resistance in these isolates was consequently not determined. A total of 67 CTX-M/pAmpC genes were found in the 35 *E. coli* isolates and 41 in the 21 *Klebsiella* spp. isolates. CTX-M was the most common gene detected in both *E. coli* (*n* = 25) and *Klebsiella* spp. (*n* = 16) followed by DHA (*n* = 15 in *E. coli* and *n* = 9 in *Klebsiella* spp.). DNA sequencing of 19 randomly selected CTX-M amplicons from *E. coli* (*n* = 13) and *Klebsiella* spp. (*n* = 6) revealed a predominance of *bla*_CTX-M-15_ (*n* = 14) and occasional detection of *bla*_CTX-M-55_ (*n* = 3), *bla*_CTX-M-3_ (*n* = 1) and *bla*_CTX-M-186_ (*n* = 1). A full list of CTX-M and pAmpC gene content in individual strains is presented in the Additional file 1: Table S1.

**Table 3:** Distribution of ESBL enzymes according to species

ESBL enzymes	*E. coli* (*n* = 35)	*Klebsiella* spp. (*n* = 21)	Total
CTX-M	28	16	44
DHA	15	9	24
MOX	10	7	17
FOX	7	5	12
CMY	7	4	11
TOTAL	104	57	161

### ERIC-PCR results for *E. coli*

**Fig. 1:**
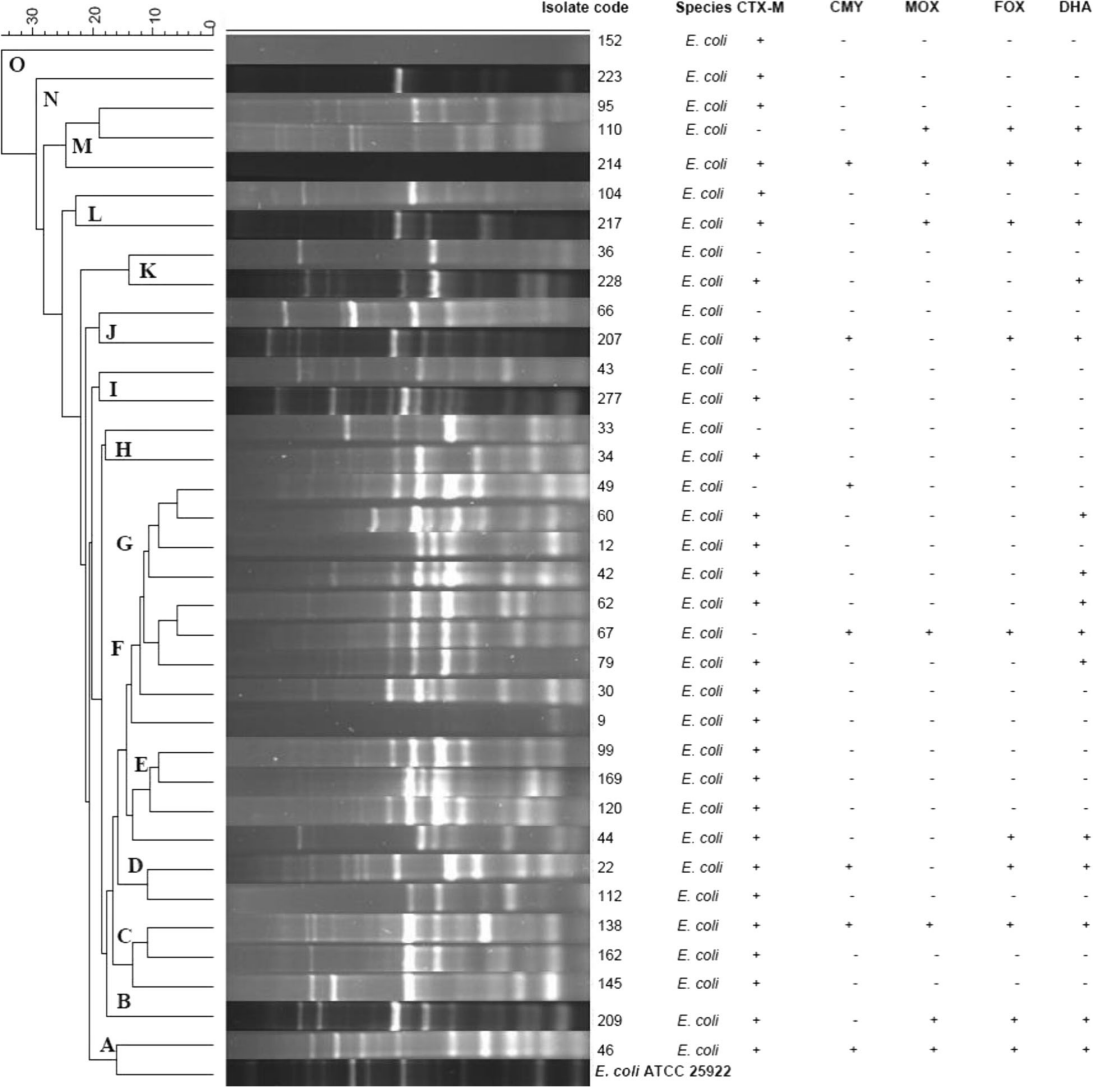
Dendrogram representing the genetic relatedness and cluster analysis of 35 *E. coli* strains isolated from stool samples of university students, based on ERIC-PCR fingerprinting patterns using Jacquard index and UPGMA algorithm. The scale at the top represents percentage similarity to *E. coli* ATCC 25922

Distinct ERIC-PCR profiles were obtained for the 35 *E. coli* isolates (Fig. 1). The absence or presence of a band was noted in determining variation among isolates, and banding patterns comprised between 2 and 14 individual bands. Fragments of different molecular weights were observed in the ERIC-PCR fingerprints, ranging from 0.5–20 kb (Fig. 1). Amplification of different intensities was observed and visual analysis of the ERIC profiles included primary, secondary and tertiary amplification (Fig. 1). Primary amplification products refer to those products of high intensity, which appear extremely bright on the gels. Secondary amplification products are those products that are not as bright as the primary amplification products but more intense that the tertiary amplification products, while the tertiary amplification products are the minor amplification products of low intensity [22]. All isolates were typeable using this fingerprinting technique and band profiles were reproducibly obtained under similar experimental conditions on repeat amplification.

The ERIC-PCR profiles allowed differentiation of the 35 *E. coli* isolates into 24 ERIC-PCR types which were grouped into 15 clusters (A-O), each of which were futher sub-divided into multiple sub-clusters (Fig. 1). CTX-M and pAmpC genes were amplified from isolates in different clusters. Isolates with similar profiles demonstrated varying beta-lactamase gene content.

### ERIC-PCR results for *Klebsiella* spp.

**Fig. 2:**
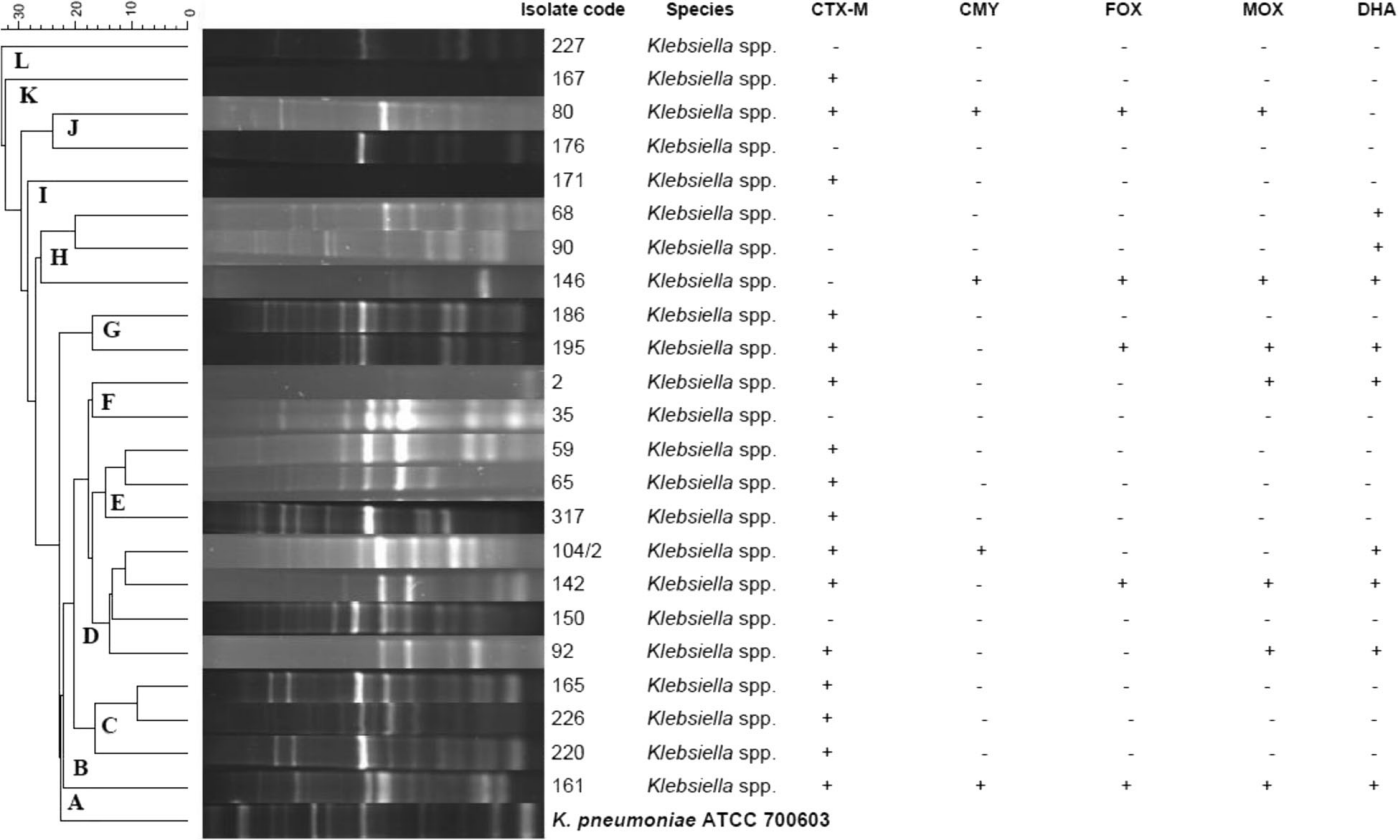
Dendrogram representing the genetic relatedness and cluster analysis of 21 *Klebsiella* spp. strains isolated from stool samples of university students, based on ERIC-PCR fingerprinting patterns using Jacquard index and UPGMA algorithm. The scale at the top represents percentage similarity to *Klebsiella pneumoniae* ATCC 700603

Twenty-one *Klebsiella* spp. isolates were subjected to ERIC-PCR analysis in comparison to the SHV-18 containing *K. pneumoniae* ATCC 700603. Distinct profiles were obtained for all isolates tested using ERIC-PCR fingerprinting (Fig. 2). Banding patterns comprised between 2 and 16 individual bands. Fragments of different molecular weights were observed in the ERIC-PCR fingerprints, ranging from 0.5–20 kb (Fig. 2).

The ERIC-PCR profiles allowed differentiation of the 21 isolates into 17 ERIC-PCR types which were grouped into 12 clusters (A-L), each of which were further sub-divided into multiple sub-clusters (Fig. 2). CTX-M and pAmpC genes were identified in isolates from different clusters, predominantly clusters B-G. Isolates with similar profiles demonstrated varying beta-lactamase gene content.

## Discussion

Antibiotic-resistant bacteria are an escalating cause of infections in Mozambique and worldwide, but information regarding the molecular epidemiology of ESBL- and pAmpC-producing Enterobacteriaceae in the country remains scarce. To our knowledge, this is the first study reporting on gastrointestinal colonization with ESBL- and pAmpC-producing Enterobacteriaceae among university students in Mozambique.

We systematically collected samples from healthy students to determine the prevalence of colonization by *E. coli* and *Klebsiella* spp. carrying ESBL and/or pAmpC, as well as their antibiotic susceptibility. In total, 20% of students (55/275) were colonized with ESBL and/or pAmpC positive *E. coli* and *Klebsiella* spp. Similar results were found in a study conducted in pre-school children attending childcare facilities in the Lao People’s Democratic Republic where the ESBL prevalence was 23% [23] while the prevalence amongst patients in intensive care in Korea was 28.2% with a higher frequency in *E. coli* (78%) compared with *Klebsiella* spp. (18%) [24]. A study recently conducted in Madagascar [18] in community settings demonstrated lower rates of colonization by ESBL-positive Gram-negative bacilli compared to this study, despite the fact that more species were included in addition to *E. coli* and *Klebsiella* spp. The present study showed a higher prevalence of ESBL and pAmpC colonization compared to a study conducted in rural communities in France where the frequency of ESBL colonization was 5.3% [25]. This large difference may be attributed to poorly controlled antibiotic consumption and sub-optimal hygiene conditions in developing countries [26]. In Mozambique, antibiotic therapy is mostly empirical because of scarce diagnostic microbiology facilities [27]. We found 36% co-existence of ESBL and pAmpC beta-lactamase genes which is high compared to reports from clinical isolates in Turkey (13.9%) [28].

The CTX-M positive isolates carried different genotypes; *bla*_CTX-M-15_ (*n* = 14), *bla*_CTX-M-55_ (*n* = 3), *bla*_CTX-M-186_ (*n* = 1) and *bla*_CTX-M-3_ (*n* = 1). These results are similar to findings in a study conducted in Tanzania [29] that found *bla*_CTX-M-15_ genes in 95% of the carriers, but differ from a study in Kenya [30] which demonstrated only 29% of the isolates carrying *bla*_CTX-M-15_ and 4% carrying *bla*_CTX-M-3_. No isolates harbored *bla*_CTX-M-55_ or *bla*_CTX-M-186_ in the Kenyan study. *bla*_CTX-M-55_ is endemic in many Asian countries, and the detection of this variant in Mozambique may reflect travel and trade with this part of the world [31].

In this study of healthy students, none of the participants had a history of hospitalization 6 months prior to the study, and 87.5% had not consumed any antibiotics at least 3 months prior to the study. This suggests that the high antibiotic resistance rates are not related to antibiotic consumption or hospitalization, and may indicate that the *E. coli* and *Klebsiella* ESBL- and pAmpC-producers isolated in this study were acquired in the community. In the university residences there is a mixture of students from different courses including medicine and health sciences, but the study was not powered to analyse differences between student groups (data not shown). ESBL- and/or pAmpC-colonized students working in health institutions may constitute a reservoir for further spread of multi-resistant microorganisms among patients.

To control the rapid dissemination of resistant Enterobacteriaceae among students and, consequently, the general population, it is necessary to educate students about the importance of personal hygiene. It is also necessary to perform further studies to determine the prevalence of colonization in different groups of the population. The prevalence in different studies may vary depending on socio-economic status of individuals involved [32], and this makes it difficult to estimate the prevalence in the general population in Mozambique. One may suppose that transmission between students is facilitated because of the conditions they live under with many individuals sharing the same bathroom and kitchen. However, the diversity of strains demonstrated using ERIC-PCR indicates that there is at present no widespread clonal outbreak, although there could be horizontal transfer of plasmids or other mobile genetic elements. One may speculate that students are exposed to ESBL and/or pAmpC isolates from some external source like dissemination in the food supply. Very little is known about the occurrence of ESBL and pAmpC strains in the food supply in Mozambique, but it is reported from other countries that ESBL and pAmpC *E. coli* and *Klebsiella* spp. may disseminate in food animals and environmental sources [33–37].

## Conclusions

This study demonstrated a 20% prevalence of colonization by multi-drug resistant, non-clonally-related ESBL- and/or pAmpC positive *E. coli* and *Klebsiella* spp. isolates among male and female university students in Mozambique. Colonization of ESBL/pAmpC in the population of study was not related to prior antimicrobial consumption or hospitalization. Similar studies should be done to further explore the epidemiology of multi-drug resistant Enterobacteriaceae in the population as a whole.

## Additional file

Additional file 1: **Table S1**. Resistance genes identified and sensitivity results of *E. coli* and *Klebsiella* spp. (DOCX 38 kb)

## Abbreviations

ATCC: American Type Culture Collection
DNA: Deoxyribonucleic acid
ERIC-PCR: Enterobacterial repetitive intergenic consensus PCR
ESBL: Extended-spectrum beta-lactamases
pAmpC: Plasmid-mediated AmpC beta-lactamase
PCR: Polymerase chain reaction
TAE: Buffer containing Tris base, acetic acid and EDTA (Ethylenediaminetetraacetic acid)
UPGMA: Unweighted pair group method with arithmetic mean
