# Supplementary material for: Faecal colonization of E. coli and Klebsiella spp. producing extended-spectrum beta-lactamases and plasmid-mediated AmpC in Mozambican university students
Source: BMC Infect Dis. 2018 May 30;18:244. doi: 10.1186/s12879-018-3154-1 (PMC5975407; doi:10.1186/s12879-018-3154-1)
Supplement: Supplementary file 1 — Table S1. Resistance genes identified and sensitivity results of E. coli and Klebsiella spp. (DOCX 38 kb) [file 12879_2018_3154_MOESM1_ESM.docx]

Table S1 Resistance genes identified and sensitivity results of *E. coli* and *Klebsiella* spp.

| Isolate | Species | ESBL genotype | | | | |  | Antibiotic susceptibility* | | | | | | | |
| --- | --- | --- | --- | --- | --- | --- | --- | --- | --- | --- | --- | --- | --- | --- | --- |
|  |  | CTX-M | CMY | FOX | MOX | DHA |  | FOX | CIP | CAZ | GEN | TET | CRO | SXT | IMP |
| 2 | *Klebsiella* spp. | CTX-M-3 | - | - | + | + |  | S | R | I | S | R | R | R | S |
| 9 | *E. coli* | + | - | - | - | - |  | S | I | R | S | R | R | R | S |
| 12 | *E. coli* | + | - | - | - | - |  | S | S | R | S | R | R | R | S |
| 22 | *E. coli* | + | + | - | + | + |  | R | R | I | S | R | R | R | S |
| 30 | *E. coli* | CTX-M-15 | - | - | - | - |  | I | R | R | S | S | R | I | S |
| 33 | *E. coli* | - | - | - | - | - |  | S | S | S | R | S | R | S | S |
| 34 | *E. coli* | CTX-M-15 | - | - | - | - |  | S | S | I | R | R | R | R | S |
| 36 | *E. coli* | - | - | - | - | - |  | S | R | R | S | R | R | R | S |
| 42 | *E. coli* | + | - | - | - | + |  | S | S | R | S | S | R | S | S |
| 43 | *E. coli* | - | - | - | - | - |  | S | S | R | S | R | R | R | S |
| 44 | *E. coli* | CTX-M-15 | - | - | + | + |  | R | S | R | S | R | R | R | S |
| 46 | *E. coli* | CTX-M-15 | + | + | + | + |  | R | R | R | R | R | R | R | S |
| 49 | *E. coli* | - | + | - | - | - |  | R | R | R | S | S | R | R | S |
| 59 | *Klebsiella* spp. | + | - | - | - | - |  | R | S | R | S | I | R | S | S |
| 60 | *E. coli* | + | - | - | - | + |  | S | S | R | S | R | R | S | S |
| 62 | *E. coli* | + | - | - | - | + |  | R | S | R | S | S | R | S | S |
| 65 | *Klebsiella* spp. | + | - | - | - | - |  | S | S | S | R | R | R | R | S |
| 66 | *E. coli* | - | - | - | - | - |  | S | S | R | S | R | R | R | S |
| 67 | *E. coli* | - | + | + | + | + |  | R | R | R | S | I | R | S | S |
| 68 | *Klebsiella* spp. | - | - | - | - | + |  | S | S | S | S | R | R | R | S |
| 79 | *E. coli* | CTX-M-15 | - | - | - | + |  | S | S | R | S | R | R | R | S |
| 80 | *Klebsiella* spp. | + | + | + | + | - |  | R | R | R | S | R | R | R | S |
| 90 | *Klebsiella* spp. | - | - | - | - | + |  | S | R | I | S | R | R | R | S |
| 92 | *Klebsiella* spp. | + | - | - | + | + |  | R | S | S | R | R | R | R | S |
| 95 | *E. coli* | + | - | - | - | - |  | S | S | S | S | R | R | R | S |
| 99 | *E. coli* | CTX-M-15 | - | - | - | - |  | S | S | R | S | R | R | R | S |
| 104 | *E. coli* | CTX-M-15 | - | - | - | - |  | I | R | R | S | S | R | I | S |
| 104/2 | *Klebsiella* spp. | + | + | - | - | + |  | R | S | R | S | I | R | S | S |
| 110 | *E. coli* | - | - | + | + | + |  | R | S | S | S | R | R | S | S |
| 112 | *E. coli* | + | - | - | - | - |  | S | S | S | S | R | R | R | S |
| 120 | *E. coli* | + | - | - | - | - |  | S | I | R | S | S | R | I | S |
| 138 | *E. coli* | CTX-M-15 | + | + | + | + |  | R | R | R | S | R | R | R | S |
| 142 | *Klebsiella* spp. | CTX-M-55 | - | + | + | + |  | R | R | R | R | R | R | R | S |
| 145 | *E. coli* | CTX-M-186 | - | - | - | - |  | I | R | R | S | S | R | I | S |
| 146 | *Klebsiella* spp. | - | + | + | + | + |  | R | S | I | S | R | R | S | S |
| 150 | *Klebsiella* spp. | - | - | - | - | - |  | S | S | I | S | S | R | R | S |
| 152 | *E. coli* | + | - | - | - | - |  | S | S | S | S | S | R | S | S |
| 161 | *Klebsiella* spp. | CTX-M-15 | + | + | + | + |  | R | R | R | R | R | R | R | S |
| 162 | *E. coli* | CTX-M-15 | - | - | - | - |  | S | S | I | S | S | R | R | S |
| 165 | *Klebsiella* spp. | CTX-M-15 | - | - | - | - |  | S | I | R | R | R | R | R | S |
| 167 | *Klebsiella* spp. | CTX-M-55 | - | - | - | - |  | R | I | R | S | S | R | I | S |
| 169 | *E. coli* | CTX-M-15 | - | - | - | - |  | S | S | R | S | R | R | R | S |
| 171 | *Klebsiella* spp. | + | - | - | - | - |  | S | S | S | R | R | R | R | S |
| 176 | *Klebsiella* spp. | - | - | - | - | - |  | S | S | S | S | R | R | R | S |
| 186 | *Klebsiella* spp. | + | - | - | - | - |  | R | I | R | S | S | R | I | S |
| 195 | *Klebsiella* spp. | + | - | + | + | + |  | R | I | R | R | R | R | R | S |
| 207 | *E. coli* | + | + | - | + | + |  | R | I | R | S | R | R | R | S |
| 209 | *E. coli* | CTX-M-15 | - | + | + | + |  | R | R | R | R | R | R | R | S |
| 214 | *E. coli* | + | + | + | + | + |  | R | R | R | R | R | R | R | S |
| 217 | *E. coli* | + | - | + | + | + |  | R | S | R | S | R | R | S | S |
| 220 | *Klebsiella* spp. | + | - | - | - | - |  | S | I | R | R | R | R | R | S |
| 223 | *E. coli* | + | - | - | - | - |  | S | R | S | S | R | R | R | S |
| 226 | *Klebsiella* spp. | + | - | - | - | - |  | S | S | S | R | R | R | R | S |
| 228 | *E. coli* | CTX-M-15 | - | - | - | + |  | R | R | R | S | R | R | R | S |
| 277 | *E. coli* | + | - | - | - | - |  | S | S | S | S | S | R | S | S |
| 317 | *Klebsiella* spp. | CTX-M-55 | - | - | - | - |  | S | R | I | S | R | R | R | S |

*S=susceptible, I=intermediately susceptible, R=resistant, FOX=cefoxitin, CIP=ciprofloxacin, CAZ=ceftazidime, GEN=gentamicin,TET=tetracycline, CRO=ceftriaxone, SXT=cotrimoxazole, IMP=imipenem.
